# Foraging Behavior Shows Individual-Consistency Over Time, and Predicts Range Use in Slow-Growing Free-Range Male Broiler Chickens

**DOI:** 10.3389/fvets.2022.814054

**Published:** 2022-02-07

**Authors:** Vitor Hugo Bessa Ferreira, Arthur Simoni, Karine Germain, Christine Leterrier, Léa Lansade, Anne Collin, Sandrine Mignon-Grasteau, Elisabeth Le Bihan-Duval, Elodie Guettier, Hélène Leruste, Hanne Løvlie, Ludovic Calandreau, Vanessa Guesdon

**Affiliations:** ^1^JUNIA, Comportement Animal et Systèmes d'Elevage, Lille, France; ^2^INRAE, CNRS, IFCE, Université de Tours, Centre Val de Loire UMR Physiologie de la Reproduction et des Comportements, Nouzilly, France; ^3^Department of Physics, Chemistry and Biology, IFM Biology, Linköping University, Linköping, Sweden; ^4^INRAE, UE EASM, Le Magneraud, Surgères, France; ^5^INRAE, Université de Tours, BOA, Nouzilly, France

**Keywords:** behavioral consistency, domestic bird, free-range chickens, personality, welfare

## Abstract

Recent research on free-range chickens shows that individual behavioral differences may link to range use. However, most of these studies explored individual behavioral differences only at one time point or during a short time window, assessed differences when animals were out of their social group and home environment (barn and range), and in specific tests or situations. Therefore, it is yet unclear how different behaviors relate to range use and how consistent these behaviors are at the individual level. To fill this gap, we here aimed to describe the behavioral budget of slow-growing male broiler chickens (S757N) when in their social group and home environment during the whole rearing period (from the second week of life to the twelfth week, before slaughter), and to relate observed behavioral differences to range use. For this, we followed a sample of individuals in two flocks (*n* = 60 focal chickens out of 200 chickens per flock), over two seasons, during three periods: before range access (from 14 to 25 days old), during early range access (first weeks of range access, from 37 to 53 days old), and during late range access (last weeks of range access, from 63 to 87 days old). By the end of each period, individual tests of exploration and social motivation were also performed, measuring exploration/activity and sociability propensities. Our results show that foraging (i.e., pecking and scratching at the ground) was the only behavior that correlated to range use for all three rearing periods, independent of the season. Foraging was also the only behavior that showed within-individual consistency from an early age and across the three rearing periods. Foraging may, therefore, serve as a useful behavioral predictor of range use in free-range broiler chickens. Our study increases the knowledge of how behaviors develop and relate to each other in a domesticated and intensely selected species, and improves our understanding of the biology of free-range broiler chickens. These findings can, ultimately, serve as a foundation to increase range use and improve chicken welfare.

## Introduction

In free-range systems, commercial domestic fowl (*Gallus gallus domesticus*), such as broiler chickens and laying hens, are allowed access to an outdoor open space, beyond the barn, known as the range. The range is often considered as a type of environmental enrichment where animals can express natural behaviors, such as foraging, locomotion, and dust bathing (1–3). In this area, animals may express a larger behavioral panel, and have their individual needs fulfilled to a higher degree, compared to animals kept indoors (4–6). Free-range chickens are also expected to have improved control over what they do and their choices, such as exploring the range or deciding to stay in the barn, which may contribute to improvement of their welfare (7, 8). However, a remaining issue with this type of system is the inconsistent range use among individuals from the same flock (9, 10). Indeed, some chickens are known to never leave the barn, while others are responsible for most of the range visits within a flock (11). As evidenced by different studies on free-range laying hens, this situation can cause health and behavioral problems due to a high density of individuals in the barn (e.g., rapid litter deterioration, increased level of parasitism, increased indoor temperature, increased aggressive pecking behavior, and underuse of provided enrichment) (12–16).

Earlier studies investigating the variable range use in commercial chickens focused mainly on differences between flocks (i.e., environmental/extrinsic factors, such as season or the presence of trees) or intrinsic individual factors, such as strain (slow vs. fast growing), sex (male vs. female), or age (young vs. old) (17–20). With increasing age, for example, the number of slow-growing broiler chickens outside, visiting and exploring the range, also increases (20, 21). However, these results do not fully explain why flocks submitted to the same environmental conditions and with limited genetic variation still present substantial variation in range use between individuals (11, 22).

To understand range use variability, an essential factor to consider is that chickens differ in personalities [i.e., individual behavioral differences that are consistent over time and across situations (23, 24)]. As a consequence, individuals may differ also in their cognitive styles, in other words, may have different ways of perceiving, interacting with, and responding to their physical and social environments (25, 26). Recent research on free-range chickens (laying hens and broiler chickens combined) provide converging evidence that individual behavioral and cognitive differences may be linked to range use (3, 27–35). For example, behaviorally, low-ranging (fast and slow-growing) broiler chickens seem to be more anxious, fearful, and less prone to forage for their feed compared to their high-ranging flock-members (3, 22, 36). Cognitively, low-ranging, slow-growing broiler chickens performed better than high-ranging ones during spatial memory and inhibitory control tasks (27, 28, 30). These differences may be explained by differences in underlying physiology: high-ranging, fast-growing broilers chickens, when subjected to manual handling and restraint, have lower plasma corticosterone concentrations than low-ranging ones (22).

Most of the mentioned studies, including ours (3, 27–30), explored behavioral and cognitive differences of free-range laying hens and broiler chickens at only one time point, or during a short time window, and, generally, used individuals that had already experienced the range. We recently showed that 3-week-old slow-growing broiler chicks without prior experience of access to a range showed a negative association between individual social motivation and the use of the range (28), while older broilers (11–14-week-old), with prior experience of a range, had the inverse pattern (29). These apparent contradictions may have arisen for two possible reasons. First, these individual differences were assessed when broilers were out of their social group and home environment (barn and range), thus in specific tests and situations (3, 27–30). While this approach can be useful to identify a link between specific behavioral patterns (such as fearfulness and social motivation) and range use, the unfamiliar settings during the tests may produce behavioral responses different from those shown in familiar environments, such as the range (37, 38). Second, the contradictions can arise because, during ontogeny, individual behavioral patterns can change (39, 40). For example, domestic and feral chickens are highly sociable early in life, but social reliance weakens when animals get older (41, 42).

To better understand individual behavioral differences when chickens are in their familiar group and home environment, the behavioral consistency over ontogeny, and how different behaviors relate to range use, we described, in the current work, the behavioral budget of slow-growing broiler chickens in their familiar group and home environment during the whole rearing cycle (from the second week of life to the twelfth week, before slaughter) and related observed behavioral patterns to range use during this time. Behavioral budgets were recorded in a sample of birds in two different flocks of male free-range broiler chickens (*n* = 60 chickens per flock), followed over two seasons (one flock in spring, and one during fall), and over three different periods: before range access (2–3-week-old), during early range access (5–7-week-old, first weeks of range access), and during late range access, before slaughter (9–12-week-old, last weeks of range access). At the individual level, we assessed how several behaviors at different rearing periods were related to range use. In addition, behavioral tests were performed at the same three mentioned rearing periods to measure individual variation in exploration/activity and sociability propensities of individual chickens.

Since the range is an environment where chickens are expected to explore, variation in explorative behaviors, such as foraging and environment pecking, was expected to be positively linked to range use. Furthermore, since chickens are a social species, we expected social motivation to correlate negatively with range use during early periods (before range access), but positively during later periods (after range access), as shown in our previous works (28, 29). Concerning the behavioral consistency over ontogeny, we predicted that behaviors of free-range chickens would be less consistent within individuals (i.e., more flexible) for early than for late periods of observation, similar to what was found for red junglefowl (39), the ancestor of domestic chicken.

## Methods

### Ethical Statement

This study was conducted at the experimental unit UE 1206 EASM (https://doi.org/10.15454/1.5572418326133655E12) of INRAE, France, from February to May (spring) and October to December (fall) 2019. It was conducted under the INRAE ethics committee approval (APAFIS #17824-2018112611585147 v4 and APAFIS #21240-2019061811063005 v3) in agreement with the French legislation.

### Animals and Housing

A full description of the husbandry procedures is described in (3, 27–30). For the two flocks used (spring and fall, 2019) in this study, we followed the same procedures. In short, each flock was composed of two hundred naked-neck (S757N) male broiler chickens (*Gallus gallus domesticus*) reared from their first day of life to 12 weeks of age in a free-range system with a stocking density of eight individuals/m^2^ in the barn (4.85 x 5.15 m) and 0.4 individuals/m^2^ in the outdoor range (27.3 x 18 m). The chickens had free access to their range beginning from 36 days of age to slaughter (around 12 weeks of age). The range was a meadow-like, open space with vegetal cover, without trees or shelters.

Inside, continuous artificial lighting was provided during the first 3 days after arrival from the hatchery. From 4 to day 14, lights were gradually decreased until reaching a natural light-dark cycle. The indoor ambient temperature was maintained at 28 °C during the first week and decreased by 1°C each week until it reached 23°C when the birds were 38 days old. Indoor, birds had *ad libitum* access to food and water.

Between days 7 and 9, all 400 chicks (200 per flock) were individually marked with a unique wing tag. Among these 400 animals, 120 chicks (60 per flock) were randomly selected and simultaneously identified via a rectangular yellow plastic poncho around the neck with unique acronyms, for easy identification (3, 27–30).

### Focal Behavioral Observations (Home Environment Condition)

As soon as chicks were set up in the barn (one day old), one observer (AS) stayed in the presence of the animals for ≥3 h per day, to habituate them to his presence and noises of the stopwatch. This habituation procedures lasted for seven days.

**Figure 1:**
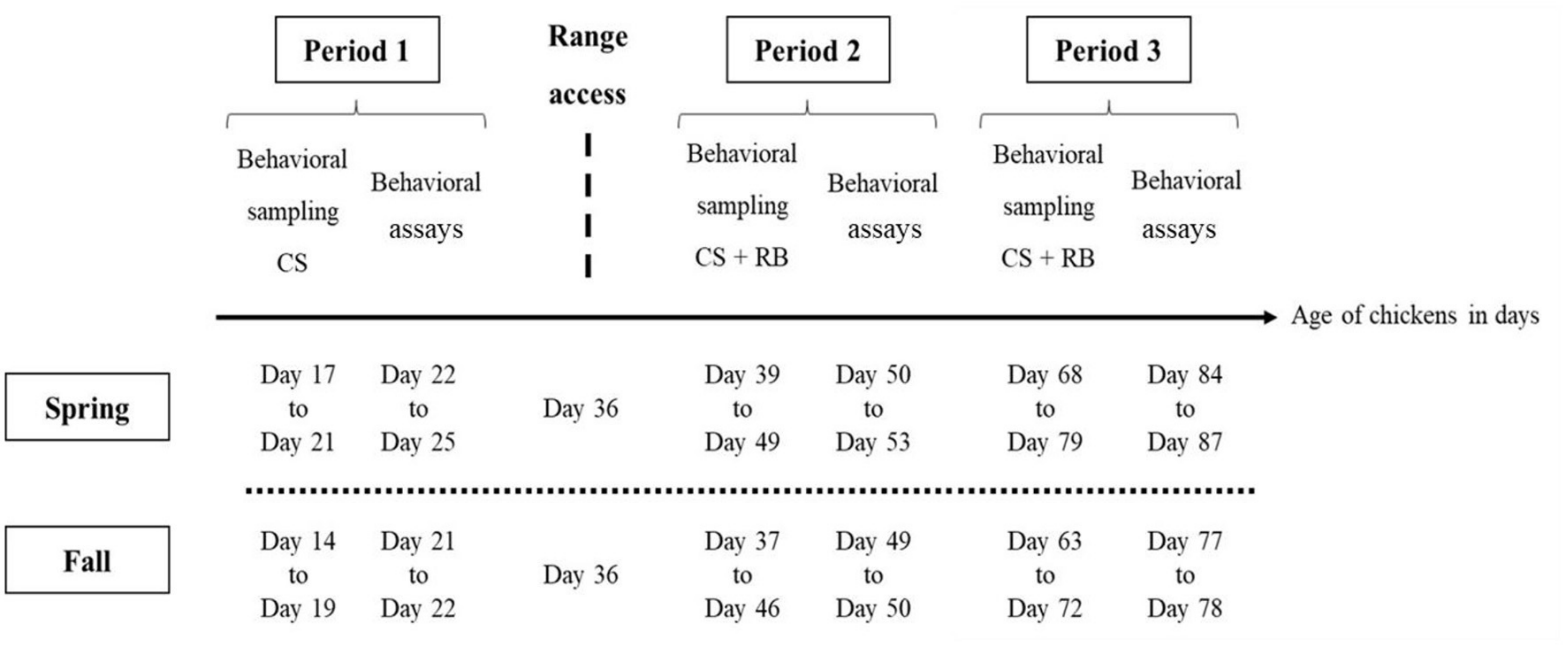
Schedule of behavioral observations (Continuous sampling, CS), Ranging behavior measurements (RB), and behavioral assays (exploration and social motivation tests) carried out during the production cycle of free-range chickens. Period 1 comprises of 5 and 6 days of behavioral observations, and 4 and 2 days of behavioral assays, before range access, for spring and fall, respectively. Period 2 and 3 comprise each, 5 days of behavioral observations and ranging behavior measurements (scan sampling) and 4 and 2 days of behavioral tests, for spring and fall, respectively. Range access was given when chicken were 36 days-old.

Over the two seasons (*n* = 60 chickens per season), in the home environment (in the barn and on the range), one experimenter (AS) observed the behavioral budget of the 60 individually marked individuals, over three different periods. In France, slow-growing free-range chickens under “Label Rouge” or “Organic Farming” labeling have a minimum slaughter age of 81 days (~12 weeks). Three rearing periods were therefore chosen to cover the whole rearing cycle and to investigate the ontogeny of behavior (39). The first period (period 1) represented the time before range access (between day 17 and 21, during spring, and between day 14 and 19, during fall). The second period (period 2) represented the first weeks of range access (day 39–49, during spring, day 37–46 during fall). Thirdly, the last time period (period 3) represented the last weeks of range access (from day 68 to 79, during spring, and from day 63 to 72, during fall, Figure 1).

Observation days were continuous over period 1, while for periods 2 and 3, observations occurred 3 days per week, interspaced by 1, or 2 days. Each observation day started at sunrise, when light conditions allowed for animals to be easily identified (between 6:30 and 9:30 in the morning), and ended by sunset (between 17:00 and 20:00, which depended on the season). During spring, observations on the same day were interspaced by two, or two and a half hours. During fall, as days shortened over time, observations within the same day were spaced from 3 hours (period 1) to 2 hours (periods 2 and 3).

To obtain the behavioral budget of chickens and rate of recorded behaviors, behavioral data were collected *via* a continuous sampling method. Each focal animal was observed for 30 s, five times a day during the spring, and four times a day during the fall. Behaviors were divided into “states” and “events.” “States” were all behaviors lasting more than 1 s (e.g., standing, resting, and locomotion), and “events” were those lasting <1 s (e.g., shaking, wing flapping and pecking). An event could also occur in “bouts” (a short period of intense activity of a specific behavior), but events within bouts were counted separately and individually (3, 43–45). For example, if an individual pecked 10 consecutive times, each peck was singly counted as an event, independent of the time interval between pecks. Events were recorded in an “all occurrences” sampling method, meaning that every event behavior was counted within the 30 s of each focal animal. The observer recorded the duration of “state” behaviors using a stopwatch and the number of occurrences of “event” behaviors. The full ethogram is described in (Table 1).

**Table 1:** Ethogram of recorded behaviors of free-range chickens (*Gallus gallus domesticus*) in their home environment (barn and range).

**Traits**	ATOL references^**^	**Descriptions**
**State behaviors**		
Standing	ATOL_0000835	Stands in an upright position, with no foot movements.
Resting	ATOL_0000837 ATOL_0000816	Sits relaxed, sleeps. The body and hocks are touching the floor.
Locomotion	ATOL_0000805	Moves for at least two or more steps without pecking or scratching the ground.
Foraging	ATOL_0000844 ATOL_0000845	Scratches or pecks the ground, and/or a plant.
Feeding-Drinking	ATOL_0002158 ATOL_0000363 ATOL_00003611	Include two behaviors: • Feeding: Feeds at the feed containers in the barn. • Drinking: Drinks at the water containers in the barn.
Social negative behaviors^*^	ATOL_0000902 ATOL_0000813	An individual fights, chases, or threatens one or multiple individuals.
Dust bath^*^	ATOL_0000824	Foot scratching and bill-raking on the floor or litter, followed by vertical wing flaps, head rubbing, and or scraping with one leg in the extended position, and finally shakes to remove dust from the plumage.
**Events behaviors**		
Comfort behaviors	ATOL_0000653 ATOL_0000822 ATOL_0000823 ATOL_0000360	Including one of five behaviors: • Shaking: Ruffles the feathers and shakes the body (not during dust bathing). • Stretching: Stretches wing or leg. • Tail wagging: Wiggles its tail horizontally or vertically. • Wing flapping: Flaps its extended wings in a vertical plane. • Preening: Grooms its plumage with the beak in a sitting, lying, or standing position.
Environment pecking	ATOL_0000845	Pecks the walls of the barn/range.
Positive social pecking	ATOL_0000846 ATOL_0000817 ATOL_0000826	Pecks on the beak of a flockmate (mainly to collect feed particles) or on another part of the body without damage to the plumage and skin of the targeted chicken

* *These behaviors were rare (<5% of the observed individuals expressed the behavior) and were discarded from statistical analyses*.

** *Traits in reference to the ontology ATOL: https://www.atol-ontology.com/en/atol-2/, in accordance with the PILLOW project data management plan*.

Individual behaviors were recorded over 15 days during spring (5 days for each period), and 16 days during fall (6 days in period 1, and 5 days for period 2 and period 3), resulting in 37.5 min of observation per chicken during the spring, and 32 min of observation per chicken during the fall, for all 120 males.

### Behavioral Assays

Behavioral assays followed the same procedures as described in (28). During spring and fall, all marked chickens underwent two behavioral tests (exploration test, and social motivation test) at three different ages: 22–25 days, 50–53 days, and 84–87 days during spring, and 21–22 days, 49–50 days, and 77–78 days during fall. The two tests were performed in two separate testing rooms by the same two experimenters (AS and VHBF). Animals were randomly assigned to be tested either in the morning (between 08 and 12 h) or in the afternoon (between 14 and 17 h), and in the exploration test or the social motivation test first. Chickens were tested individually in both tests, and submitted to only one test per day. For each individual, tests were carried out over two consecutive days. The tested animals were observed directly by an out-of-view experimenter, using a digital video camera recorder connected to a monitor.

Before behavioral assays, birds were captured and kept in crates (74.5 × 54.5 × 29 cm, maximum of eight to four individuals/crate, with reducing numbers of individuals as birds grew larger) without food, for at least 30 min for acclimatation to the crates/testing room. After testing, to prevent re-catching of tested chickens, individuals were kept in another crate with the same dimensions and stocking density. At the end of each half-day (~4 h for all individuals to be tested), all tested individuals were released back into the barn.

#### Exploration Test

The exploration propensity of an individual was measured when exposed to a novel environment or arena (40, 46–48). The test arena was a square enclosure with opaque walls and a vinyl floor divided into 16 marked equal-sized areas. The size of the arena varied between the three different periods to limit the effect of habituation to the test arena and to accommodate the increased size of the birds (39) (Period 1: 1 × 1 × 2 m; Period 2: 1.2 × 1.2 × 2 m; Period 3: 1.6 × 1.6 × 2 m). Across periods, different floor substrates (Period 1: straw, Period 2: wood shavings, and Period 3: bare vinyl) were used to maintain novelty and minimize habituation over periods (39). An empty feeder, identical to those present in the barn, thus familiar to all birds, was placed in the center of the arena to partially obstruct the individuals' field of vision and encourage animal locomotion and exploration (39).

During all test periods, after the 30 min of acclimatation to the crates/testing room, and in order to minimize the effect of any fear linked to the exposure to a new environment, chickens were initially placed in groups of four in the arena for 10 min. Once all chickens had undergone this habituation stage, each was subjected to the exploration test (~90 min between the first and last individual tested, after the end of group habituation). Chickens were placed individually at the same starting point (a corner of the arena), and left free to explore the arena for 5 min, while behavior was recorded. The recorded behaviors were the number of zones visited (at least more than half of the animal body present in the zone) and time spent in foraging behavior (i.e., the animal scratches or pecks the ground) (28).

#### Social Motivation Test

Social motivation tests are used to measure the degree of sociability of an individual (24, 46, 49, 50). The test arena we used was a rectangular corridor with wooden walls and a vinyl floor, divided into five marked, equal-sized areas. The arena was covered with a wire mesh to prevent individuals from escaping. Again, the size of the arena varied according to the three test periods: 1 × 0.4 × 0.7 m during period 1; 1.25 × 0.5 × 0.7 m during period 2, and finally 1.5 × 0.6 × 0.7 m during period 3. Similar to the exploration test, different floor substrates (Period 1: straw, Period 2: wood shavings, and Period 3: bare vinyl) were used, and the animals again underwent a short habituation within the arena before being tested. In the spring, individuals were habituated for 10 min in groups of four, while during the fall, the group habituation lasted 15 min.

After habituation, each bird was individually placed in a closed and dark box at one end of the corridor for 30 s. After this time, manually operated guillotine door was opened so that the animal had free access to the corridor for 5 min. Three randomly chosen familiar conspecifics (from the same flock, but not focal individuals) were located at the end of the corridor and visible to the focal individual. These familiar conspecifics were changed every four individuals tested. The observed behavior was the time spent in the two zones closest to the conspecifics (measured in seconds).

### Ranging Behavior

To determine the number of individual range visits by each of the focal chickens (i.e., their ranging behavior), we followed the same procedures as previously described by (3, 27–30). Briefly, the experimenters (AS and VHBF), using binoculars, counted the marked birds on the range from a high chair placed outside the range, to minimize disturbance. Seven interspaced scans per day during spring, and five scans during fall (~2 h between each scan, from sunrise to sunset) were performed. A range visit was counted when a chicken had their two feet outside of the barn. Our previous research has shown that the number of range visits is positively correlated with how far chickens go from the barn (3, 27–30). The scan measurements were performed for 10 days over periods 2 and 3 (when chickens could access the range), before and after the behavioral budget observations.

## Statistics

Through behavioral sampling, we computed the behavioral budget (recorded as “states”) and rate of behaviors (recorded as “events”) for each chicken. The duration of each state behavior was divided by the total time of observation and multiplied by 100, which gave the time allocated in percentage. The total number of each event behavior was divided by the total hours of observation (0.62 h per individual during spring, and 0.53 h per individual in fall), to calculate the rate of these behaviors (number of events/hour).

Since the number of scans to obtain the number of individual range visits was greater in spring compared to fall (seven scans per day in spring compared to five scans per day during fall), we divided the number of range visits by the total number of scans in each season and multiplied by 100, which gave a similar scale to compare the number of range visits between the two flocks. This was done separately for period 2 and period 3 (5 days each) and for the total of periods 2 and 3 (10 days).

Following analyses on the ontogeny and consistency of red junglefowl personality (39), our first step was to verify whether differences in the mean level of expression of behaviors were present between spring and fall of each behavior at each period using general linear modeling with repeated measures. The between-subject factor was “season” (spring, fall) and the within-subject factor were the “period” (periods 1, 2, and 3) and the “season ^*^ period” interaction. We applied Greenhouse-Geisser corrections for assumptions of sphericity when that was violated. Main effects or interactions that were significant were followed by multiple comparisons corrected with Bonferroni.

To verify any relationship between the behavior of chickens in their home environment and their behaviors during individual tests, we ran, within each period, non-parametric, partial Spearman correlations, which allowed us to control for season (spring, fall). Chicken behaviors were also correlated to the total percentage of individual range visits (% period 2 + % period 3), to determine which behaviors were linked to range use. Finally, to verify if behaviors were consistent over time, non-parametrical partial correlations, within each behavior and across periods, were run. Through these analyses, we aimed to verify whether behaviors from consecutive periods were similar (period 1 x 2, and period 2 x 3), but also whether chicken behavior after range access could be predicted by early behavior, before range access (period 1 x period 3). Due to the multiple correlations carried out, *p*-values were corrected using the Holm-Bonferroni method.

All statistical analyses were performed using IBM SPSS 21. Statistical significance was accepted at *p* ≤ 0.05 (after corrections, when applied).

During the experiment, three individuals died, one during spring and two during fall. Our sample size, therefore, consisted of 59 chickens during spring, and 58 chickens during fall.

## Results

**Figure 2:**
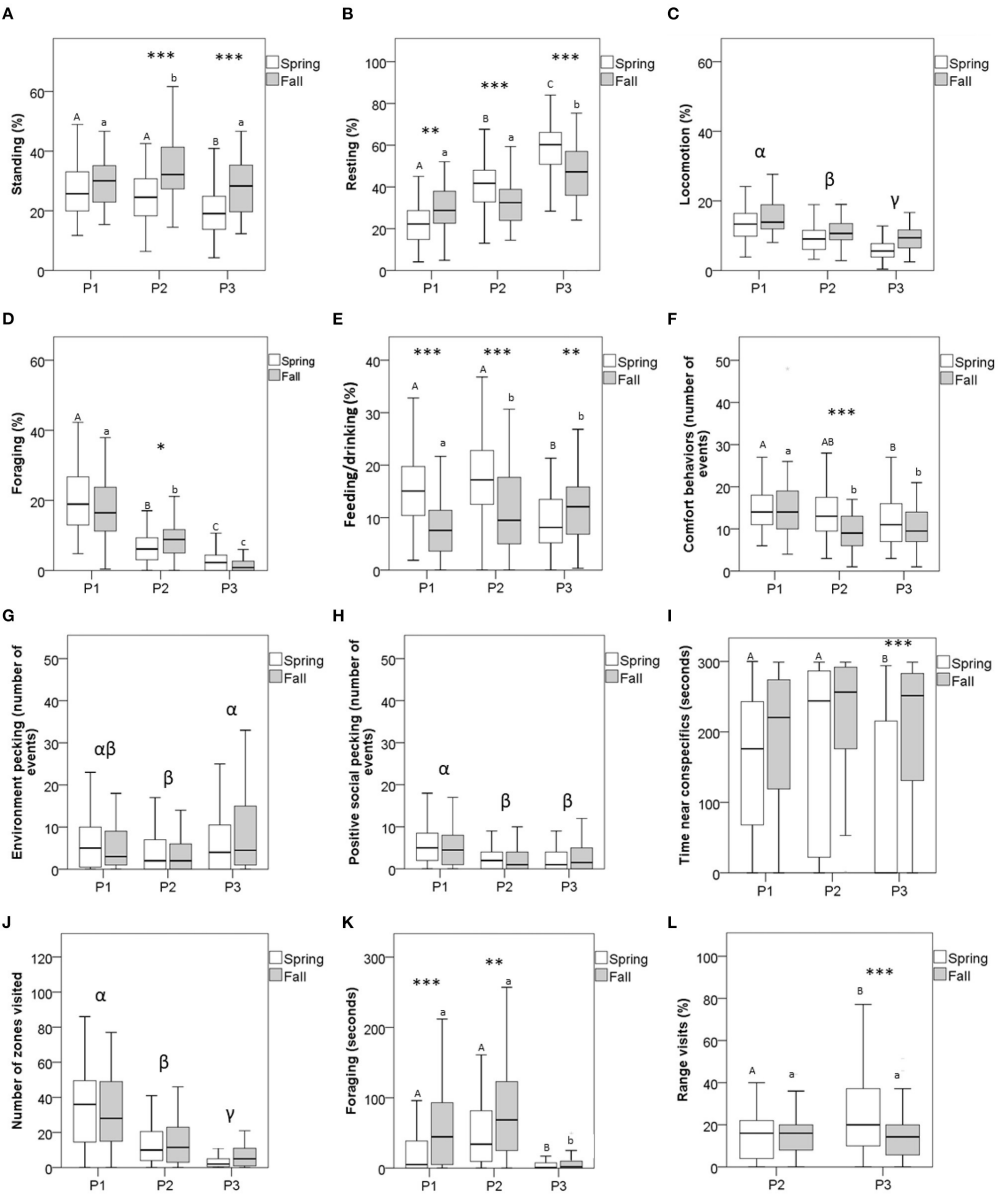
Mean expression of the behaviors of male broiler free-range chickens in both their home environment and in the test situations (Social motivation and exploration tests) at three different periods of their ontogeny (P1 - before range access, P2 - early range access, and P3 - late range access), and two different seasons (spring and fall, *n* = 59 and *n* = 58, respectively). State behaviors are presented in **(A)** Standing, **(B)** Resting, **(C)** Locomotion, **(D)** Foraging, and **(E)** Feeding/Drinking. Events behaviors are presented in **(F)** Comfort behaviors, **(G)** Environment pecking, and **(H)** Positive social pecking. Results of tests are presented in **(I)** Time near conspecifics, **(J)** Number of zones visited, **(K)** Foraging, and **(L)** Number of range visits. Asterisks indicate significant differences between the seasons at each period. *p < 0.05, **p < 0.01 level, ***p < 0.001 (General linear modeling with repeated measures). Uppercase letters indicate significant differences between periods in spring. Lowercase letters indicate significant differences between periods in fall. Asterisks indicate significant differences between seasons and within period. Greek letters indicate significant differences between periods. Data are presented as median and percentiles.

Significant interactions between season and period were present for most of the recorded behaviors (7 out of 11, Table 2). For these 7 behaviors, a difference between spring and fall was observed for at least one of the three observed periods (Figure 2). Some of these differences seemed to be occasional, such as for foraging (which was lower during spring than fall, but only in period 2), comfort behaviors (which was higher during spring than fall, again in period 2), time spent near conspecifics during the social motivation test (which was lower during spring than fall, only in period 3), and the number of range visits (which was higher during spring than fall, only in period 3). For other behaviors, such as standing, resting, and feeding/drinking, differences between seasons varied across periods (Table 2 and Figure 2). Locomotion was lower during spring than fall, and decreased over periods similarly for both seasons. Positive social pecking and the number of zones visited during the exploration test both decreased over periods, while environment pecking remained stable over time (Table 2 and Figure 2).

**Table 2:** Statistics on the effects of period, season, and their interactions on the mean-level expression of behaviors of free-range chicken in their home environment and in the individual tests (social motivation, SM, and exploration tests, ET).

	**Period**	**Season**	**Period × Season**
Standing	F_2,230_ = 12.94 *p* < 0.001	F_1,115_ = 35.49 *p* < 0.001	F_2,230_ = 5.51 *p* = 0.005
Resting	F_2,230_ = 176.41 *p* < 0.001	F_1,115_ = 11.22 *p* = 0.001	F_2,230_ = 21.72 *p* < 0.001
Locomotion	F_2,230_ = 99.12 *p* < 0.001	F_1,115_ = 24.35 *p* < 0.001	F_2,230_ = 1.14 *p* = 0.32
Foraging	F_1.53, 176.01_ = 253.15 *p* < 0.001	F_1,115_ = 0.64 *p* = 0.42	F_1.53, 176.01_ = 5.61 *p* = 0.009
Feeding/drinking	F_2,230_ = 11.04 *p* < 0.001	F_1,115_ = 19.06 *p* < 0.001	F_2,230_ = 20.42 *p* < 0.001
Comfort behaviors	F_2,230_ = 12.16 *p* < 0.001	F_1,115_ = 10.63 *p* = 0.001	F_2,230_ = 5.26 *p* = 0.006
Environment pecking	F_2,230_ = 5.31 *p* < 0.001	F_1,115_ = 0.27 *p* = 0.6	F_2,230_ = 2.67 *p* = 0.07
Positive social pecking	F_1.13, 129.97_ = 1531.44 *p* = 0.003	F_1,115_ = 1.27 *p* = 0.26	F_1.13, 129.97_ = 1.04 *p* = 0.31
Time spent near conspecifics (SM)	F_2,230_ = 8.01 *p* < 0.001	F_1,115_ = 16.54 *p* < 0.001	F_2,230_ = 4.76 *p* = 0.009
Number of zones crossed (ET)	F_1.43, 165.02_ = 110.39 *p* < 0.001	F_1,115_ = 0.31 *p* = 0.57	F_1.43, 165.02_ = 1.32 *p* = 0.26
Foraging (ET)	F_1.68, 193.82_ = 50.53 *p* < 0.001	F_1,115_ = 0.87 *p* < 0.001	F_1.68, 193.82_ = 5.23 *p* = 0.009
Range visits	F_1,115_ = 10.40 *p* = 0.002	F_1,115_ = 7.18 *p* = 0.008	F_1,115_ = 12.12 *p* = 0.001

*Degrees of freedom, F- and p-values are given (General linear modeling with repeated measures). Greenhouse-Geisser corrections were applied on degrees of freedom when assumptions of sphericity were violated*.

Although the behavioral budget and rate of behaviors varied within each period between seasons, the analyses of behavioral changes over time (between periods and within each season) showed that, overall, mean-level behavioral patterns were not stable across time. Most behaviors decreased with age for both seasons (Table 2 and Figure 2). Resting was the only behavior that increased over time for both seasons (Table 2 and Figure 2). Other behaviors, such as comfort behaviors, showed different patterns between seasons, either by being consistent for all periods within one season and not by the other, or by following opposite directions (increasing during spring, and decreasing during fall, for example, such as standing and the time near conspecifics, Table 2 and Figure 2).

None of the behaviors we recorded in the home environment were correlated with behaviors obtained in individual tests (the exploration test, or the social motivation test; Table 3). Foraging was the only behavior that showed positive correlations with the number of range visits along all periods (Table 3).

**Table 3:** Rank correlations between behaviors of male broiler free-range chickens in their home environment and in the individual tests (social motivation, SM, and exploration tests, ET), over different periods.

	**Period**	**Period 1/Period 2/Period 3**			**Period 2 + Period 3**
		**Time spent near conspecifics (SM)**	**Number of zones crossed (ET)**	**Foraging (ET)**	**Range visits**
Period 1	Standing	−0.12	−0.08	−0.09	−0.01
	Resting	−0.05	−0.06	0.02	−0.12
	Locomotion	0.09	0.01	0.04	−0.07
	Foraging	0.12	0.03	0	**0.31**
	Feeding/drinking	0	0.14	0.02	−0.14
	Comfort behaviors	−0.09	−0.07	−0.01	−0.16
	Environment pecking	−0.04	−0.08	−0.11	0.01
	Positive social pecking	−0.05	0.1	−0.11	−0.02
	Time spent near conspecifics (SM)	-	0.12	0.02	0.23
	Number of zones crossed (ET)	0.12	-	−0.07	−0.01
	Foraging (ET)	0.02	−0.07	-	−0.03
Period 2	Standing	−0.06	−0.08	−0.04	−0.02
	Resting	−0.05	−0.07	−0.06	−0.2
	Locomotion	−0.14	−0.09	0.07	0.06
	Foraging	0.18	0.13	−0.16	**0.42**
	Feeding/drinking	0.11	0.14	0.11	−0.01
	Comfort behaviors	−0.05	−0.06	0.03	0
	Environment pecking	0.03	−0.17	−0.02	−0.17
	Positive social pecking	−0.16	−0.18	0	−0.19
	Time spent near conspecifics (SM)	-	0.10	−0.19	0.18
	Number of zones crossed (ET)	0.10	-	−0.01	0.01
	Foraging (ET)	−0.19	−0.01	-	0.01
Period 3	Standing	−0.11	−0.09	−0.1	−0.08
	Resting	0.11	0.07	0.14	−0.18
	Locomotion	−0.05	0.06	−0.04	0.06
	Foraging	−0.05	0.03	0	**0.4**
	Feeding/drinking	−0.02	−0.06	−0.06	0.05
	Comfort behaviors	0.16	0.05	0.08	0.18
	Environment pecking	0.07	0	0	0.01
	Positive social pecking	−0.12	0.11	0.04	−0.07
	Time spent near conspecifics (SM)	-	0	−0.01	0.13
	Number of zones crossed (ET)	0	-	0.21	0.09
	Foraging (ET)	−0.01	0.21	-	0

*Periods were considered as follows: Period—before range access, Period 2—early range access, and Period 3—late range access. Significant partial Spearman rank correlation coefficients are indicated in bold (p < 0.05)*.

Foraging was also the only behavior that showed individual consistency over all periods (Table 4). Over time, between periods 2 and 3, more behaviors became consistent, which was the case for resting, the time spent near conspecifics during the social motivation test, the number of zones visited and foraging during the exploration test, and the number of range visits (Table 4).

**Table 4:** Consistency (rank correlations) of different behaviors of male free-range broiler chickens over different periods.

		**Rank correlation for a given behavior between periods**		
	**Behaviors**	**Period 1–Period 2**	**Period 2–Period 3**	**Period 1–Period 3**
Home Environment	Standing	0.12	0.20	0.11
	Resting	0.14	**0.31**	−0.06
	Locomotion	0.17	0.06	0.16
	Foraging	**0.27**	**0.22**	**0.32**
	Feeding/drinking	0.14	0.12	−0.05
	Comfort behaviors	0.09	−0.07	−0.01
	Environment pecking	0.02	0.08	0.11
	Positive social pecking	0.02	0.07	−0.06
	Range visits	**-**	**0.38**	**-**
Tests	Time spent near conspecifics (SM)	0.11	**0.22**	0.06
	Number of zones crossed (ET)	0.11	**0.31**	0.08
	Foraging (ET)	0.14	**0.31**	−0.06

*Behaviors were observed both at the home environment and during individual tests (social motivation, SM, and exploration tests, ET). Periods were considered as follows: Period 1—before range access, Period 2—early range access, and Period 3—late range access. Significant partial Spearman rank correlation coefficients are indicated in bold (p < 0.05)*.

## Discussion

This is the first study to our knowledge that followed the individual behavior of slow-growing free-range broiler chickens in an exhaustive manner along the animals' full productive cycle. To this aim, behaviors were recorded both in the chickens' home environment and under standardized test conditions, over two different seasons (spring and fall). Our principal objective was to better understand how several behaviors relate to range use and how consistent these behaviors were over different rearing periods, before and after chickens could access the range. Although both observation period and season impacted chicken behavior at the group level, at the individual level, we confirmed that behavioral variation in foraging (i.e., pecking and scratching at the ground) was positively linked to range use. Chickens that foraged more (during all periods) used the range more, which suggests an individual characteristic that underlies individual motivation to use the range. Additionally, foraging was the only behavior that showed a significant consistency before and after range access, while other behavioral variables (such as resting, the time spent near conspecifics during the social motivation test, foraging and the number of zones crossed during the exploration test) became consistent only after range access, showing that, similarly to its wild ancestor (39), behavioral consistency of free-range chickens can appear early during the individual development for some, but not all, behaviors. While these results are promising for a better understanding of range use of slow-growing free-range broiler chickens, they are based on just two flocks, in two different seasons. The following discussion requires, therefore, a careful interpretation.

Since chicken behaviors are strongly influenced by photoperiodic cycles (51), it was not surprising that differences in behaviors occurred between seasons. Indeed, our results agree with data on broiler breeder hens. During summer, hens spent more time resting and eating but less time in locomotion, compared to hens during winter (52). In our work, the percentage of range visits did not differ between seasons during the first weeks, but did so during the last weeks of range access. Chickens increased range visits over time during spring but decreased range visits during fall. Corroborating other studies on free-range broiler chickens, longer days and warmer temperatures likely motivate chickens to go outside the barn. In comparison, shorter days and cooler temperatures may have the opposite effect (10, 18).

Occasional differences between seasons for specific rearing periods are less easily explained. As an example, chickens during fall seemed to be more sociable based on their behavior during the social motivation test: they spent more time near conspecifics when close in age to slaughter, compared to chickens tested during the spring. One possibility for these results is that, over days, weather gets colder in the fall, causing chickens to be closer to get warm, and warmer in the spring, causing chickens to disperse to avoid heat stress. However, caution is needed with the interpretation of these results since only two flocks were compared. Therefore, more studies are needed to verify if these results are based on a real seasonal effect, and better understand the real implications of these variations.

Along studied periods, free-range broiler chickens gradually diminished their time on energetically demanding behaviors (such as foraging and locomotion), and allocated more time to less costly behaviors (such as resting). This finding is in accordance with the theory of resource allocation (53, 54). This theory predicts that when selection promotes certain expensive behaviors or biological processes, the energy for other demanding behaviors/processes must decrease. In the case of domestic fowl, the need to forage, or to move to collect environmental information, may be less important than the need to direct energy for growth, which can significantly reduce foraging, locomotion and the number of zones visited in the exploration test in broiler chickens bred for meat production (55).

As typically done in animal personality studies, we also investigated the relationship between behaviors in home environment and variables obtained during individual tests, to verify if they measure the same behavioral propensities (46). We could not confirm any relationship, as no significant correlations were found. For chickens, being totally or partially isolated (such as during the exploration test, or during the social motivation test, respectively) is not a common or natural situation, which can result in behavioral responses (e.g., anxiety or fear) different from those that specific tests are intended to measure (37, 38). Besides isolation, catching and crating of chickens may have also influenced our results, acting as a stressful experience before behavioral tests. To counteract these influences, the individual identification *via* the plastic ponchos was a way to facilitate the spotting and capturing of chickens, which in turn reduced the time of manipulation, and minimized stress effects. Furthermore, our previous results showed that differences between low-ranging and high-ranging chickens are restricted to few and specific behavioral aspects, such as foraging behavior, social motivation, and complex cognitive domains (3, 28–30). Although we consider there was no general effect of stress on our tested individuals, future individual tests need to be adapted to the biology of domestic chickens in order to effectively link the behaviors expressed during the tests to those expressed in the home environment (56).

However, when evaluating the relationship between behavioral variables and range use (number of range visits), we did find positive and significant correlations between and range use and foraging behavior: in the home environment, foraging, a wellknown exploratory behavior in chickens, was the only behavior that correlated with range use for all three rearing periods, independent of the season. This result corroborates other studies in free-range laying hens and broiler chickens showing that the range is a zone for exploratory behaviors, such as foraging and pecking (1, 10). Interestingly, in our study, chicks' foraging, before range access (in the barn), correlated positively with later range use. Foraging may then serve as a predictive behavioral marker of range use, and it should be further explored if encouraging the foraging behavior of animals from an early age would increase also their use of the range. Indeed, for laying hens, the use of forage sources was the most successful method on-farm to attract birds into the range compared to providing shelterbelts and artificial shade (57). Providing forage sources continuously within the first weeks of the life of chickens in the barn may increase their propensities to express this particular behavior in the range, when they finally have access to it. A promising research avenue is to investigate the genetics behind this behavior, how fixed it is (i.e., whether it has high heritability), and how gene-environment interactions influence the relationship between foraging and range use.

Consistency of behaviors was surprisingly similar to what was found for the ancestor of domestic chickens, the red junglefowl. During the chick period, red junglefowl showed consistency for foraging and exploration behaviors (39). For the junglefowl, exploration was recorded as the number of crossed zones in a test arena, which can have similarities to our measure of free-range chickens' foraging behavior in their home environment: although the range becomes familiar over time, it is a novel environment during the first weeks of range access. Therefore, for chickens to forage in the range they need to explore this new environment and visit its different zones.

Also similar to the results on the red junglefowl, the number of consistent behaviors increased over ontogeny (39), indicating that although chickens cannot reach further developmental stages (such as sexual maturity and adulthood) due to the limitations imposed by the productive system, the rapid weight gain and development of these animals may impose a faster establishment of certain aspects of their personality. The combination of our results suggests that chickens' foraging behaviors and range use could be part of an exploratory personality axis. This is based on these two behaviors correlating and showing individual consistency over time and in different contexts and situations (such as seasons), fulfilling the necessary criteria to be considered as a personality trait (38, 58).

Curiously, neither feeding/drinking nor any event behaviors (i.e., behaviors lasting <1 s) showed individual consistencies across rearing periods. This is probably because, for feeding/drinking, access to the range may offer an alternative feed source, which may potentially disrupt the consistency of feeding/drinking at the barn (59). Indeed, intake of grass biomass in the range may represent between 2.5 and 4.5% on a dry matter basis, or 18 to 26% on a fresh basis of the total feed intake in free-range broiler chickens (60, 61). This intake may therefore reduce the need for feed and drink in the barn, disrupting our measures of behavioral consistency. Event behaviors may also be more labile, and due to their short duration, be less related to energy-expenditure/energy-saving as the state behaviors studied, causing them to be less consistent over time.

To conclude, we here followed free-range broiler chickens throughout their whole lives, from an early age, before range access, until the last weeks of life, before slaughter. During this time, we observed behaviors both in home environment (barn, range), and in individual tests (measuring exploration and social motivation). Our results show that chick foraging in the barn may be a useful predictor of range use along different rearing periods (before range access, early range access, and late range access) and seasons (spring and fall) studied. Additionally, foraging showed within-individual consistency from an early age and across the three rearing periods studied here. If responsive to environmental stimulation, this behavior should thus be promoted to maximize the use of the range by chickens in free-range systems. Combined, our results suggest that chickens' foraging behaviors and range use may be part of the same personality axis, since these two exploratory behaviors correlate with each other, and show individual consistency over time and across situations. Free-range broiler chickens can, therefore, be an interesting species to study the ontogeny of behaviors further, since these animals, due to artificial selection processes, have a rapid development and can be followed from their very first days of life until the end of their lives. Finally, our study is important in both theoretical and practical aspects. Theoretically, it increases our knowledge of how behaviors develop and relate to each other in a domesticated and intensely selected species. Practically, it provides essential data on which chicken behavioral propensities underlie range use and allows for a better understanding of free-range broiler chickens, serving as a foundation to improve chicken welfare.

## Data Availability Statement

The original contributions presented in the study are included in the article/Supplementary Material, further inquiries can be directed to the corresponding authors.

## Ethics Statement

The animal study was reviewed and approved by the INRAE Ethics Committee (APAFIS #17824-2018112611585147 v4 and APAFIS #21240-2019061811063005 v3).

## Author Contributions

VF, AS, KG, LC, and VG: conceived and designed the experiments. VF and AS: performed the experiment. VF, AS, LC, and VG: analyzed the data. VF, AS, KG, CL, LL, AC, SM-G, EL, EG, HLe, HLø, LC, and VG: wrote/reviewed the paper. All authors reviewed the manuscript.

## Funding

This experiment was partially funded by JUNIA ISA and the French National Research Institute for Agriculture, Food, and Environment (INRAE). The project PPILOW has received funding from the European Union's Horizon 2020 research and innovation programme under grant agreement N°816172, http://www.ppilow.eu.

## Conflict of Interest

The authors declare that the research was conducted in the absence of any commercial or financial relationships that could be construed as a potential conflict of interest.

## Publisher's Note

All claims expressed in this article are solely those of the authors and do not necessarily represent those of their affiliated organizations, or those of the publisher, the editors and the reviewers. Any product that may be evaluated in this article, or claim that may be made by its manufacturer, is not guaranteed or endorsed by the publisher.
